# Influence of Fuels, Weather and the Built Environment on the Exposure of Property to Wildfire

**DOI:** 10.1371/journal.pone.0111414

**Published:** 2014-10-31

**Authors:** Trent D. Penman, Luke Collins, Alexandra D. Syphard, Jon E. Keeley, Ross A. Bradstock

**Affiliations:** 1 Centre for Environmental Risk Management of Bushfires, Institute of Conservation Biology and Environmental Management, University of Wollongong, NSW, Australia; 2 Hawkesbury Institute for the Environment, University of Western Sydney, Penrith, NSW, Australia; 3 Conservation Biology Institute, La Mesa, California, United States of America; 4 U.S. Geological Survey, Western Ecological Research Center, Sequoia-Kings Canyon Field Station, Three Rivers, California, United States of America; 5 Department of Ecology and Evolutionary Biology, University of California Los Angeles, Los Angeles, California, United States of America; CSIRO, Australia

## Abstract

Wildfires can pose a significant risk to people and property. Billions of dollars are spent investing in fire management actions in an attempt to reduce the risk of loss. One of the key areas where money is spent is through fuel treatment – either fuel reduction (prescribed fire) or fuel removal (fuel breaks). Individual treatments can influence fire size and the maximum distance travelled from the ignition and presumably risk, but few studies have examined the landscape level effectiveness of these treatments. Here we use a Bayesian Network model to examine the relative influence of the built and natural environment, weather, fuel and fuel treatments in determining the risk posed from wildfire to the wildland-urban interface. Fire size and distance travelled was influenced most strongly by weather, with exposure to fires most sensitive to changes in the built environment and fire parameters. Natural environment variables and fuel load all had minor influences on fire size, distance travelled and exposure of assets. These results suggest that management of fuels provided minimal reductions in risk to assets and adequate planning of the changes in the built environment to cope with the expansion of human populations is going to be vital for managing risk from fire under future climates.

## Introduction

Wildfires can pose a significant risk to people and property [1]–[4]. Large losses of property and life have been recorded from individual fires or fire complexes in fire prone regions throughout the globe [5]–[7]. Such events can impact individuals and communities for many years [8]–[11]. As a result, fire management agencies invest significant budgets in reducing the risk of loss from wildfires, primarily through investment in fuel management and active fire suppression [12], [13].

Fuel management is a commonly used risk management tool in fire prone landscapes [14], [15]. The justification for the approach derives from the fundamentals of fire behaviour with a reduction in fuel loads expected to result in a subsequent lowering of the fire intensity and rate of spread [16]–[20]. In turn, these changes to fire behaviour are expected to increase the probability of successfully containing the fire with active fire suppression [21], [22].

Fuel breaks are a commonly applied type of fuel management treatment in a variety of ecosystems. These are mechanical reductions or removal of fuel, typically as linear features along ridgetops, to enable safe access for fire suppression crews to manage fires [23]. Empirical analysis has found fuel breaks are more effective when readily accessible and well-maintained and when used for *backfire* operations [23], [24]. Under these conditions the intensity and rate of spread are lower and containment of the fires through active suppression is more likely to be successful [21], [22], [24]. Simulation studies have found individual fuel breaks have the potential to reduce the size and intensity of wildfires [25], [26]. While studies examining the impact of fuel breaks on the behaviour of individual fires are valuable, to quantify the extent to which fuel breaks reduce risk to lives and property, we need to examine the role of fuel breaks at the landscape scale [27]. Although simulation studies have shown management of fuels can alter fire regimes in forested ecosystems, there is a need to quantify how fuel breaks, in particular, can reduce the risk of exposure of assets. Here we examine the performance of fuel breaks in mitigating risk in Mediterranean shrubland (chaparral) landscapes.

Fuel breaks are the main fuel treatment carried out in the chaparral shrublands of southern California, with a long history of extensive deployment [23], [24]. Thus case studies of their effectiveness in mitigating risk can provide valuable insight into mitigation of risk in a region with some of the highest exposure of fire-prone urban and peri-urban developments in the world. Case studies in this context may also be valuable for assessing fuel treatment options in other fire-prone, temperate environments which share similar elements of the problem [28].

Bayesian Networks (BN) provide a suitable methodology for the analysis of risk management problems [29]–[31]. They are depicted as directed acyclic graphs with nodes representing the variables and arrows representing the directional relationships between nodes [32]. There is a conditional probability table for each node that contains the joint probability distributions for the variable [33]. Root nodes occur at the top of the model and are not influenced by other variables in the model. These nodes have a conditional probability table containing a single probability for each state in that node. Child nodes are variables that are influenced by one or more variables (parent nodes). These nodes have a conditional probability table that represents the probability of a given state in the child node given the state(s) in the parent node(s). Uncertainty is propagated throughout the model, providing probability distribution for all output nodes. Results are likelihoods that form the basis for risk management calculations [29]. Bayesian Networks have been found to be a valuable method for examining fire risk management problems at the landscape scale [31], [34].

Here we develop a Bayesian Network model to examine the role of fuel breaks in reducing the risk of assets being exposed to wildfire using San Diego county as a case study area. San Diego county has a history of major fire losses (circa 5000 houses destroyed between 2000 and the present), which reflects extensive and rapidly growing developments, exposure to regular episodes of severe fire weather and terrain and vegetation conducive to the rapid spread of intense fires [35]. Thus the county comprises encapsulates key elements and a significant portion of the wildfire risk problem in southern California. As a case study it therefore provides the potential for key insights into fire management that are regionally, nationally and globally significant. The BN model combines data from a fire simulation model (FARSITE) and environmental data. We specifically seek to determine the extent to which risk posed by wildfires to properties at the wildland urban interface is influenced by the environment (weather, fuel moisture, natural environment), developmental patterns (built environment) and fuel management (fuel load, fuel breaks).

## Methods

**Figure 1:**
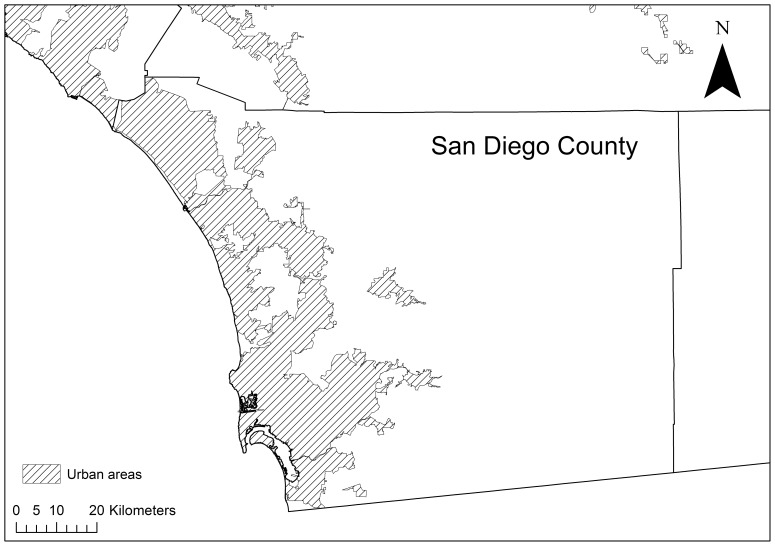
Location of the study area, San Diego County, USA.

The study area was San Diego County, California, USA, which supports a population of approximately 3.2 million people in an area of approximately 11 000 km^2^ (US Census, http://quickfacts.census.gov/qfd/states/06/06073.html, Accessed December 2013). In the county, there is a long and complex wildland urban interface [36], along which thousands of homes have been destroyed in major fire events in the last decade [37]. Native vegetation of the area is dominated by chaparral, coastal sage scrub, and oak woodland. The county experiences a Mediterranean climate with hot dry summers and winter rainfall with moderate temperatures. Fires in the county occur most frequently in summer months, but most area burned occurs in the autumn when annual fuel moisture is lowest and Santa Ana winds are most frequent [38]. San Diego County was selected as it is dominated by highly flammable shrubland vegetation and falls within known Santa Ana wind corridors, making the region prone to recurrent large fire events [39], [40]. The simulation landscape was defined as a 60 km×60 km area east of San Diego (Figure 1).

A Bayesian Network model was used to examine the relative influence of weather, the built and natural environment and fuel breaks on the risk of exposure to wildfire. Here we broadly follow the methods for developing Bayesian Networks recommended by Marcot *et al.* [41] and Chen and Pollino [42]. The primary steps used were to construct a conceptual model of the problem, develop influence diagrams to depict the relationships of the conceptual model and finally populate all the conditional probability tables within the model.

A basic conceptual model for the study was derived from previous fire management research [31], [34]. This model assumed an ignition had occurred and predicted the subsequent spread and potential impact of the wildfire upon property, which was dependent upon environmental conditions and management decisions.

**Figure 2:**
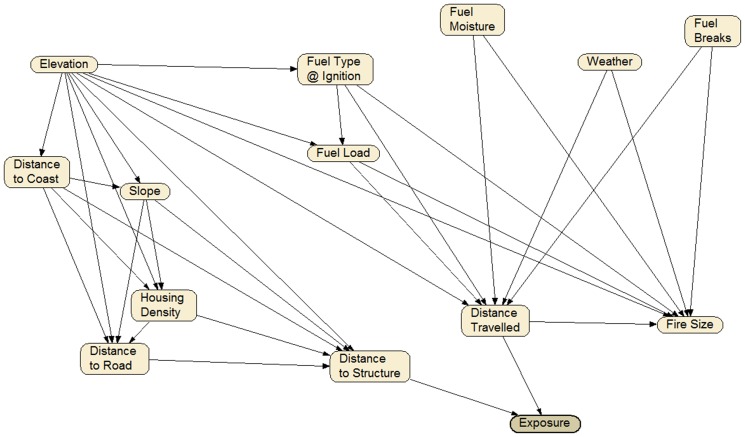
Influence diagrams for the Bayesian Network Model. See Table 1 for node definitions and states.

Influence diagrams encapsulated the conceptual framework and the relevant environmental factors (Figure 2). These were developed for the study area by the authors through a series of workshops held by the United States Geological Survey (December 2010, September 2011, May 2012) involving twelve researchers with expertise in fire management, Bayesian Network analysis and landscape ecology. Iterations of the influence diagrams were presented to the group, discussed and then further refined until a consensus was reached. In the final set of influence diagrams, fire size and distance travelled were assumed to be influenced by the key variables considered in the simulation modelling – weather, fuel moisture, landscape fuel load and the occurrence of fuel breaks within the National Forest. Elements of the natural environment at the ignition location, specifically fuel type, fuel load and elevation, were considered to have an influence on fire size and distance travelled. The built environment also influences fire spread and exposure of property. Exposure to fire was taken as simple function considering the distance the fire could potentially travel and the distance from the ignition point to property (Figure 2).

Data for the conditional probability tables in the analysis (Table 1) were derived from either a simulation study or empirical data for the study region. We undertook a comprehensive simulation of fires in the area using the Fire Area Simulator (FARSITE) using random ignition locations. FARSITE is a two dimensional spatially explicit model that models fire spread using Huygens' principle [43]. The simulations examined all combinations of fire weather (low, high and Santa Ana), live fuel moisture (LMF 60% and 90%), fuel loading (low and high) and the presence or absence of maintained fuel breaks. A total of 11,944 fires were simulated a FARSITE framework. Complete details of the approach are presented in Table S1 in Material S1. From the simulation data, we derived values for the nodes fire size and distance travelled. Weather, fuel moisture, landscape fuel load and the occurrence of fuel breaks were implemented as decision nodes to explore the relative influence of each factor. Environmental variables (i.e. elevation, ignition fuel load, ignition fuel type and distance to the interface) were derived from GIS data from the region (www.landfire.gov Accessed October 2012). All nodes and methods of discretisation are described in more detail in Table 1. The Bayesian Network model is available from the ABNMS data repository (www.abnms.org).

**Table 1:** Nodes, definitions and states used in the Bayesian Network model.

Node	Description	Levels
Distance Travelled (O)	The maximum distance the fire travelled.	0 to 0.5 km; 0.5 to 1.5 km; 1.5 to 4 km; 4 to 10 km; 10 to 15 km; >15 km
Distance to the coast (GIS)	Distance from the ignition point to the coast.	0 to 10 km; 10 to 25 km; 25 to 42 km; 42 to 68 km; 68 to 80 km; >80 km
Distance to road (GIS)	The distance from the ignition pointto the nearest mapped road.	0 to 50 m; 50 to 100 m; 100 to 500 m; 500 to 1000 m; 1000 to 2000 m; >2000 m
Distance to structure (GIS)	The distance from the ignition pointto the nearest mapped house.	0 to 50 m; 50 to 100 m; 100 to 500 m; 500 to 1000 m; 1000 to 2000 m; >2000 m
Elevation (GIS)	Elevation of the ignition point	0 to 300 m; 300 to 600 m; 6000 to 1000 m; 1000 to 4000 m; >4000 m
Exposure (C)	Are houses exposed by the fire	Yes; No
Fire size (O)	The final size of the simulated fire	0 to 20 ha: 20 to 150 ha: 150 to 1000 ha: 1000 to 5000 ha: 5000 to 10000 ha: >10000 ha
Fuel breaks (S)	Whether fuel breaks are present	Yes; No
Fuel load (S)	Landscape fuel load	High (2001); Low (2008)
Fuel moisture(S)	Live fuel moisture level at the startof the simulation	60%; 90%
Fuel type at ignition (GIS)	Broad classification of the type ofvegetation at the point of ignition.	Grass; Shrub; Tree
Housing density (GIS)	Number of houses per hectare	0 to 26; 26 to 33.5; 33.5 to 117; 117 to 205; 205 to 300; >300
Slope (GIS)	Slope under the ignition point	0 degrees; 0 to 5 degrees; 5 to 15 degrees; 15 to 30 degrees; >30 degrees
Weather (S)	Predominant conditions duringthe simulated fire	Low; High; Santa Ana

(C) = calculated variable; (GIS) = GIS derived variable; (O) = output of the simulation model; (S) = variable set in the simulation model.

Two methods were used to examine the relative influence of variables. Firstly, the relative influence of each of the modelled factors was assessed using values of the terminal node – “exposure to fire”. This node reflects the risk of property being exposed to a wildfire under the given conditions. We considered all 24 combinations of the key predictor variables – weather (3 levels), fuel moisture (2 levels), landscape fuel load (2 levels) and fuel breaks (2 levels) (Table 1). Secondly, the sensitivity of nodes was assessed using the sensitivity to findings function in Netica (http://www.norsys.com/netica.html, Accessed December 2013). This function examines the extent to which changes in one variable affects the variable of interest. We examined the sensitivity of findings for “exposure to fire” as the terminal node and “distance travelled”.

## Results

**Figure 3:**
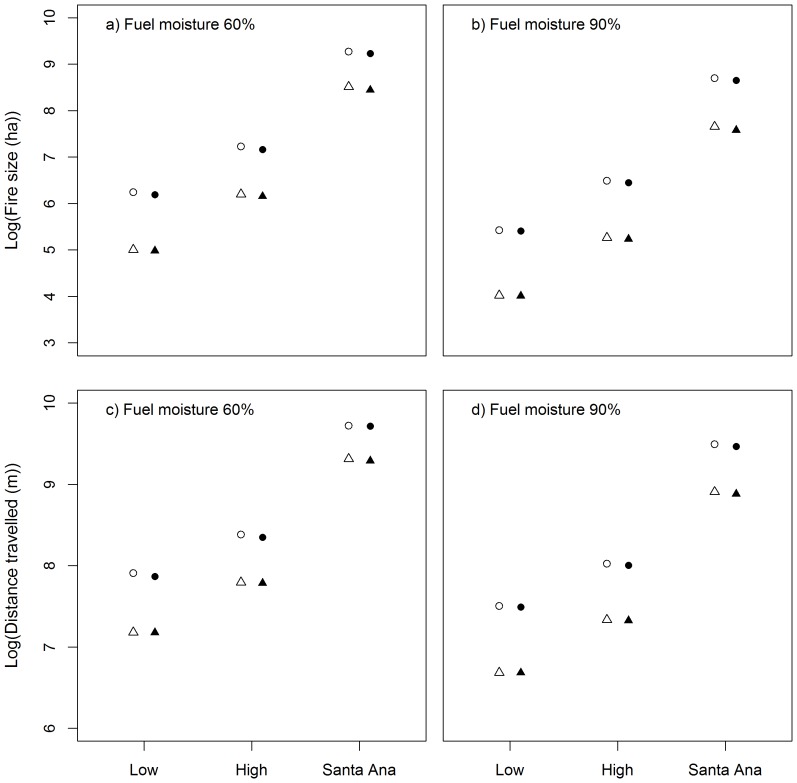
Relationships from the FARSITE simulation data between weather and fire size with fuel moisture of a) 60% and b) 90%, and distance travelled with fuel moisture of c) 60% and d) 90%. Open symbols are for simulations with no fuel breaks, closed symbols for simulations with fuel breaks. Circles represent a high landscape fuel load scenario and triangles represent a low landscape fuel load scenario. NB 95% confidence intervals were too small to depict in the graphics.

The size of fires after a 12 hour simulation ranged from 0.1 ha to 28,480 ha, with a mean of 2896 ha (±40 S.E.) and a median 882 ha. The distance travelled ranged from 18 m to 47,300 m with a mean of 6171 m (±57 S.E.). Responses of fire size and distance travelled to the predictor variables were consistent. Fire size and distance travelled increased with the severity of fire weather and the landscape fuel load, and decreased with increasing fuel moisture. The presence of fuel breaks had little influence on either individual fire size or distance travelled (Figure 3).

**Figure 4:**
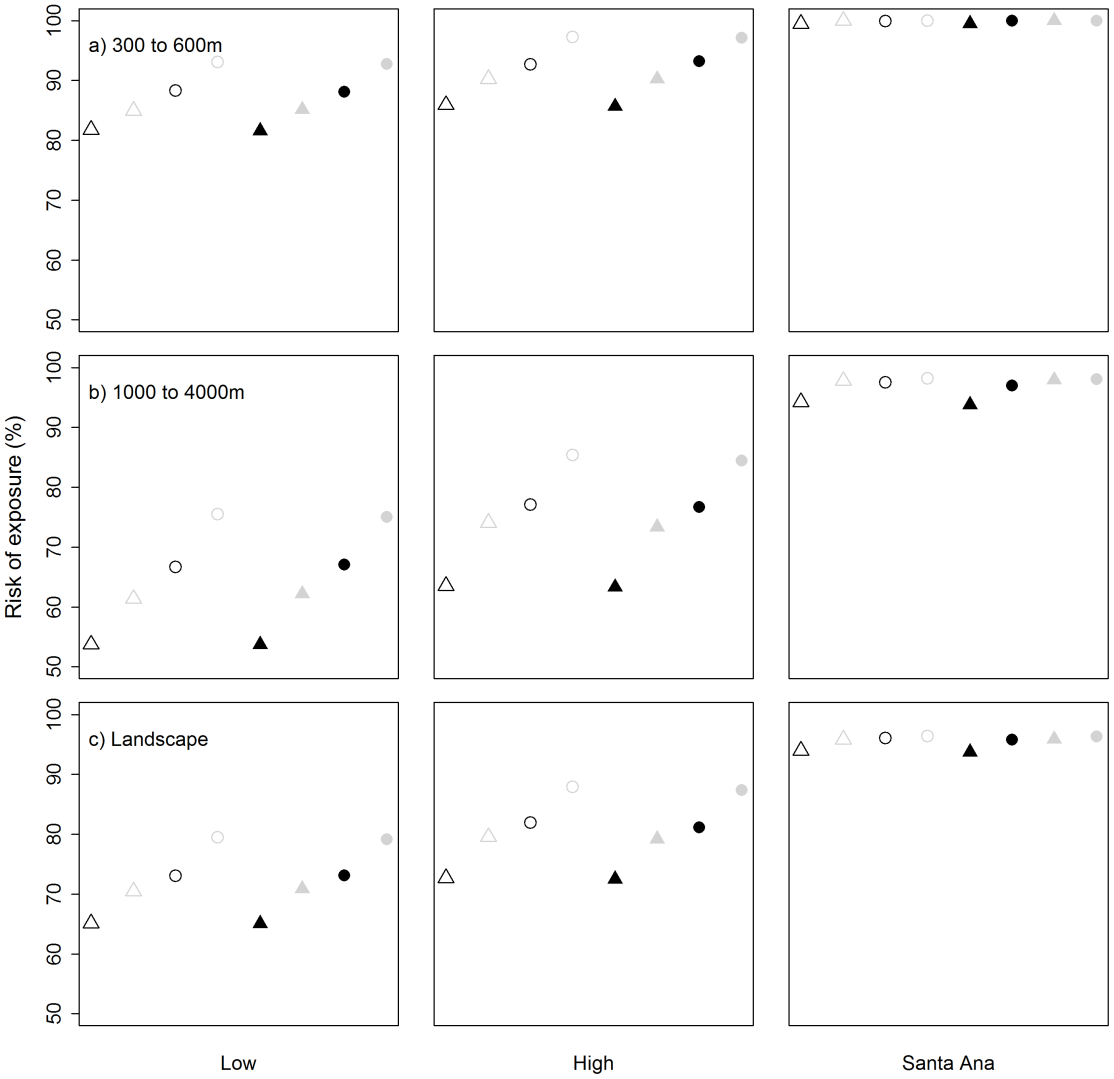
Risk of exposure for the 24 scenarios modelled for a) fires igniting at elevations of 300 to 600 m; b) fires igniting at elevations of 1000 to 4000 m; c) all locations across the landscape. Open symbols = fuel break scenarios; closed symbols = no fuel breaks; Grey symbols = fuel moisture of 60%; Black symbols = fuel moisture of 90%; Circles = high landscape fuel loads; Triangle = low landscape fuel loads.

Risk of exposure to fires was influenced most strongly by weather (Figure 4). The risk of exposure was >99% for all fuel scenarios considered under Santa Ana conditions. Under low and high weather conditions, fuel load and fuel moisture had a strong influence of risk of exposure, whereby risk was greater when landscape fuels were high compared to low and when fuel moisture was 60% compared to 90% (Figure 4). These effects were lost under Santa Ana conditions, where fuel load and fuel moisture had very little effect on the risk of exposure. Fuel breaks had very little influence on the risk of exposure. Risk varied significantly across elevation with fires starting in low elevation sites having significantly higher risk than fires starting at higher elevation sites (Figure 4).

**Figure 5:**
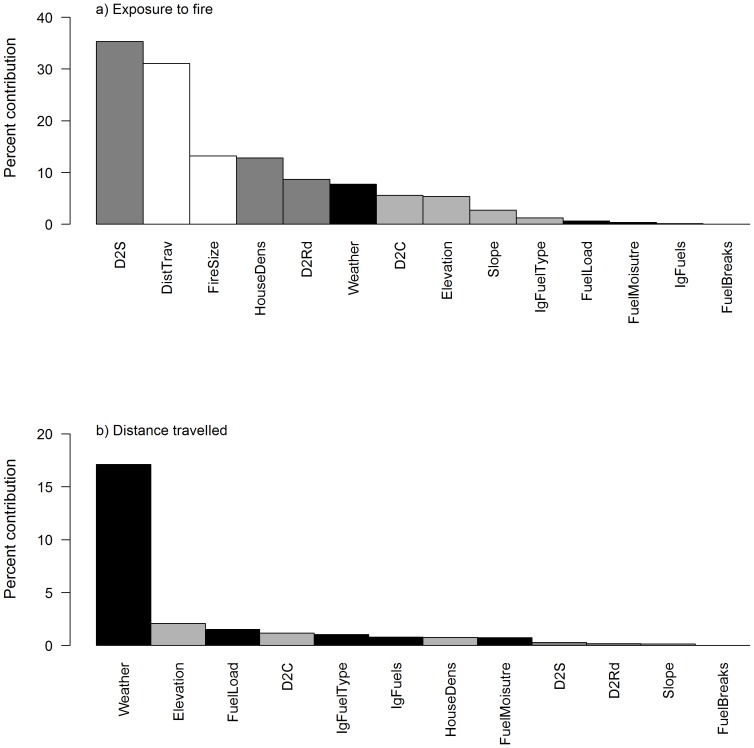
Sensitivity to findings for nodes a) Exposure to fire and b) Distance travelled. White bars = fire variables, dark grey bars = built environment variables; light grey bars = natural environment variables; Black bars = simulation model variables. D2S = distance to structure; DistTrav = distance travelled by the fire; HouseDens = housing density; D2Rd = Distance to road; D2C = distance to the coast; IgFuelType = fuel type at the point of ignition; IgFuels = fuel load at the point of ignition; FuelBreaks = presence of fuel breaks.

Exposure to fires was most sensitive to changes in the built environment, as well as fire parameters, i.e. fire size and distance travelled (Figure 5a). Distance to structures from the ignition had the strongest influence, followed by the distance travelled by a fire and the fire size. This is expected as parent nodes are likely to have the strongest influence on a node. Of the variables a greater distance from the terminal node, variables depicting the built environment (housing density and distance to road) were the next most influential variables, followed by weather. Variables describing the natural environment had only a modest influence on exposure to fires, with the fuel variables having no influence (Figure 5a). Distance travelled by a fire was primarily influenced by the weather on the day of the fire (Figure 5b). Natural environment variables and fuel load all had minor influence (<2.1%), with all built environment variables having low influence (<1%) (Figure 5b).

## Discussion

Consistent with previous research, fire size and distance travelled is most sensitive to changes in weather [18], [44]–[48] and the risk of exposure is most strongly influenced by attributes of the fire (size and distance travelled) and the nature of the built environment [34], [49]. Measured attributes of fuels had only a minor influence on fire parameters and risk of exposure. Fuel breaks in the National Forest did not affect fire size, distance travelled or the risk of exposure at the interface.

### Risk of exposure

Weather had the strongest influence on fire size and distance travelled (Figure 5b) and indirectly, the risk of exposure. It has been well documented that fire weather strongly influences fire size, the rate of spread, spotting distance, fire intensity and severity [16], [18], [40], [50]–[52]. As a result, wildfires burning under extreme conditions account for the majority of area burnt for many regions [44], [53]–[55]. Furthermore, it is under extreme fire weather conditions wildfires pose the greatest threat to people and property [50], [56]–[59].

Exposure was also influenced by the built environment, namely distance to road and housing density (Figure 5). Higher densities of properties occur at lower elevations (less than 300 m) and these have a higher risk of exposure compared with higher elevation sites independent of weather (Figure 4). Fires starting close to the interface are more likely to impact upon assets under any weather conditions [60], [61], whereas fires starting considerable distances from property are only likely to impact on property under extreme weather conditions conducive to fire spread [34], [57]. These results suggest that adequate planning of the changes in the built environment to cope with the expansion of human populations is going to be important for managing risk from fire [49], [62], [63]. We do note that the model only considers exposure to fire and we have not considered the size of the fire or the extent of the interface exposed to the fire. Fires that start away from populations will be significantly larger when they do impact on the interface compared with those that start nearby [60]. Larger fires would be expected to impact on a greater number of assets than smaller fires and these relationships require further investigation.

Of the factors relating to fuels, landscape fuel load had the strongest influence on fire size, distance travelled and risk of exposure (Figure 3; Figure 4). However, our model is more sensitive to the effects of weather and the built environment (Figure 5). Price et al. [64] found no effect of antecedent area burnt on the annual area burnt by wildfire in southern California coastal systems. The authors argue that the low effect of past fire is related to the low level of wildfire in the system (<2% per annum) and the rapid development of fuels (1–2 years). A large proportion of the study area (∼22%) burnt in wildfires during 2007 and was consequently in the early stage of fuel development (i.e. 1 year old) in the 2008 fuel layer, which may explain the strong influence of fuel load under low and high fire weather. However, in San Diego County, the majority of annual area burnt occurs under extreme Santa Ana fire weather [55] where our model found no effect of fuel load. These results support the finding of Price et al. [64] that landscape fuel treatments in these systems are unlikely to reduce the risk of fire to assets. (Figure 3; Figure 4). Scenarios with 90% fuel moisture had significantly lower risk than those with 60% under low and high conditions, but not under Santa Ana (Figure 4). Live fuel moisture is related to fire activity in southern California, with large fires generally being associated with low levels (∼60–80%) [65], [66]. Early in the fire season live fuel moisture is generally greater than 90% (Keeley et al. 2009), the resulting area burnt in southern California typically is relatively small [65], [66]. Our selection of 60% and 90% may not have truly captured the variable effect of live fuel moisture, particularly when these values exceed 100% and fire activity is expected to be low. However, the greatest risk to assets comes during Santa Ana weather conditions where there is no distinguishable effect of live fuel moisture, providing further support for our existing results. Regardless, it is typically lowest at the end of summer drought when Santa Ana winds and hence large fires are most likely [67].

Fuel breaks were ineffective at altering risk of exposure of property under any weather scenario in our study. Here we modelled fires assuming that all mapped fuel breaks in San Diego County were maintained (see Material S1; Figure 1), which exceeds current practice. Fuel breaks have been found to affect individual fire size and distance travelled [25], [26]. The network of fuel breaks in San Diego County is highly clustered (Figure 1) presumably to protect particular assets from future wildfires, although fuel breaks continue to be constructed. Clustering of the fuel breaks will result in low encounter rates with wildfires that will result in a low efficacy of this management technique [24] when considering the landscape level risk. Here we assumed a random ignition model, however ignitions do not occur randomly across landscapes [60], [68]–[70] and tend to occur close to roads and development. This is important with regards to the result that fires that start closer to homes are most likely to reach those homes. Simulations have revealed that fire size and burn probability are sensitive to the use of random against non-random ignition locations, though these biases are minimised under extreme weather conditions [71] when the greatest risk of exposure occurs (Figure 4).

The effectiveness of fuel breaks is contingent on suppression resources and access [24]. In our study, the fuel breaks were constructed in FARSITE in a manner that simulated suppression along the fuel breaks. Fuel breaks tend to be constructed to allow for control of the fire flanks and not the head fire, i.e. the point of the greatest forward rate of spread. As a result, fuel breaks are unlikely to affect the distance travelled by a fire and have negligible impacts on total fire size. Our model did not model the interaction of suppression through direct attack of the fire front or indirect attack from other breaks in the landscape, e.g. roads and rivers. Inclusion of the suppression at these points may have altered the efficacy of fuel breaks when estimating risk. Similarly, we did not consider the impact on fires of igniting backburns from fuel breaks. However, as the severity of the fire weather increases the effectiveness of suppression actions are severely diminished [22], [48], [72]. Therefore, we would only consider fuel breaks in conjunction with suppression as having potential to further reduce risk under low fire weather and not under moderate fire weather or Santa Ana conditions [24], [39].

### Fire management

Management agencies seek to reduce risk to assets acknowledging that there are no practical means to remove the risk. Weather is the primary determinate of risk to assets from fire [34], [55], [57]. While management actions can be effective under relatively benign fire weather, understanding the effectiveness of management under extreme fire weather is fundamental to determining the extent to which management can reduce risk to people and property [48]. In our model, we considered the role of fuel treatments both fuel breaks for suppression and fuel treatments through examining the role of fuel loads. Neither of these was effective under extreme fire weather despite our model considering extreme levels of fuel treatment (>20% in 1 year old fuel) and fuel breaks (all mapped breaks in the county).

A range of other fire management approaches are available that were not tested here. Three broad management areas have the potential to significantly reduce risk to assets. Firstly, ignition management to reduce the occurrence of ignitions and subsequent fires will reduce the risk to assets [44]. Included in ignition management would be rapid response or initial attack [47], [73], whereby resources are used to aggressively attempt to suppress fires before they become established fires. Secondly, improved urban planning policies to better develop the built environment to reduce the extent of the exposure [49], [62], [74]. This would include building in low risk areas outside Santa Ana wind corridors [35] and incorporating adequate offsets between vegetation and structures [74], [75]. In southern California, the best urban planning practices would be to focus on infill-type development, as low to intermediate housing density, and isolated clusters of development are the strongest risk factors for a house being destroyed in a fire [63]. Finally, reduce the vulnerability of residents and properties at the urban interface. Residents can be educated to reduce the vulnerability of their property through adequate preparation [76], [77]. Furthermore, properties can be built or retrofitted to appropriate construction standards to be more resilient to the impact of fire [78], [79]. While each of these is likely to reduce risk, only through an expanded analysis of these approaches across all weather scenarios will it be possible to identify an optimal management strategy.

## Conclusion

Weather determines the risk of exposure for assets in the landscape. Under extreme weather, where the risk of fire is greatest, landscape fuel treatments are unlikely to have a significant influence on risk. These results suggest that managing the occurrence of fire and the spatial distribution of the built environment across the landscape is likely to be the best way to alter the risk profile. Further research is needed to examine the cost trade-offs of each of these approaches.

## Supporting Information

Material S1 Supplementary text outlines the modelling process in farsite. Table S1, Fuel moisture conditions used in the simulations. Dead fuel moisture values are from Scott and Burgin (2005). See text for description of LFM categories.
(DOCX)
