# Supplementary material for: Influence of Fuels, Weather and the Built Environment on the Exposure of Property to Wildfire
Source: PLoS One. 2014 Oct 31;9(10):e111414. doi: 10.1371/journal.pone.0111414 (PMC4216070; doi:10.1371/journal.pone.0111414)
Supplement: Material S1 — Supplementary text outlines the modelling process in farsite. Table S1, Fuel moisture conditions used in the simulations. Dead fuel moisture values are from Scott and Burgin (2005). See text for description of LFM categories. (DOCX) [file pone.0111414.s001.docx]

**Supplementary material**

***Fire simulation modelling***

Fire simulations utilised the Fire Area Simulator (FARSITE). FARSITE is a two dimensional spatially explicit model that models fire spread using Huygens' principle ([Finney, 1998](#_ENREF_26)). Several models are incorporated by FARSITE to simulate fire behaviour, including surface fire spread ([Rothermel, 1972](#_ENREF_76)), crown fire spread ([Van Wagner, 1977](#_ENREF_92)) and spotting ([Albini, 1979](#_ENREF_2)). FARSITE requires the user to provide a range of input data summarising topography, fuel and weather. Topography (elevation, slope, aspect) and fuels (fuel type, canopy cover, crown height, crown base height, crown bulk density) are provided as raster grids and weather is provided as a stream of data. Weather data streams include (i) daily observations of total precipitation, temperature (maximum and minimum) and relative humidity (maximum and minimum) and (ii) sub-daily observations of wind speed, wind direction and cloud cover. Values for live fuel moisture (herbaceous and woody) and initial dead fuel moisture (1-, 10- and 100-hour fuels) are also provided by the user. Dead fuel moisture is calculated during simulations based on weather streams (i.e. fuel conditioning). Barriers to fire spread, such as roads or fuel breaks are inputted as shapefiles and incorporated by FARSITE into the simulation landscape as areas of no fuel ([Finney, 1998](#_ENREF_26)).

Ember production in FARSITE occurs when you have either active or passive crown fire ([Finney, 1998](#_ENREF_26)), and is therefore dependent on the presence of a tree canopy. Rothermel ([1972](#_ENREF_76)) surface fire spread models used in FARSITE consider shrub fuels to be part of the surface fuel complex. Consequently, there is no scope for crown fire and spotting to occur in shrubland fuel types. Spotting is an important component of shrubland fire behaviour as it allows fires to bridge both natural (e.g. water bodies) and artificial (e.g. roads, mechanical fuel breaks) impediments to fire spread ([Keeley *et al.*, 2004](#_ENREF_43)). We overcame this limitation by adding a tree canopy to tall shrublands and adjusting crown cover, bulk density, crown base height and crown height so that ember production occurred with realistic spotting distances, but without considerably altering other fire behaviour parameters (i.e. crown fire occurrence, rate of spread, fire intensity). Realistic spotting distances were considered to be maximum spotting distances up to 1600 m or more under Santa Ana conditions, with the majority of spotting distances being less than 500 – 1000 m. These values were based upon anecdotal spotting distances provided in publications (Keeley et al. 2004) and reports (The 2003 San Diego County fire siege fire safety review, [www.fs.usda.gov/Internet/FSE_DOCUMENTS/stelprdb5297020.pdf](http://www.fs.usda.gov/Internet/FSE_DOCUMENTS/stelprdb5297020.pdf), accessed 13 February 2014).

**Biophysical inputs**

Live fuel moisture (LFM) is related to fire activity in southern California, with large fires generally being associated with low LFM (~ 60 – 80 %) ([Dennison *et al.*, 2006](#_ENREF_21); [Dennison and Moritz, 2009](#_ENREF_22)). LFM content in shrubs typically falls within the 60 – 80 % range throughout a dry fire season (June - November) or towards the end of a typical fire season (October – November) ([Keeley *et al.*, 2009](#_ENREF_44)). The commencement of an average fire season (June – July) will generally see LFM values of greater than 90% ([Keeley et al. 2009](#_ENREF_7)), which are conditions under which the area burnt in southern California typically remains low ([Dennison *et al.*, 2008](#_ENREF_23); [Dennison and Moritz, 2009](#_ENREF_22)). Two LFM scenarios were examined in our study: (i) 90%, representing low fire risk conditions typical of the start of an average fire season (ALFM) and (ii) 60%, representing high fire risk conditions typical of a dry fire season or the end of an average fire season (LLFM) (Supplementary Table 1). Dead fuel moisture was varied to correspond with live fuel moisture values (Supplementary Table 1; values from Scott and Burgin ([2005](#_ENREF_78))), though fuel conditioning will alter these values prior to fire ignition (see below).

The spatial arrangement of different fuel types and successional states, particularly the connectivity of shrubland vegetation with high fuel biomass, is argued by some to be an important determinant of fire regimes across southern California ([Minnich, 1995](#_ENREF_57)). In order to account for this potential variability historical fuel layers from 2001 and 2008, which represent years when shrublands with high fuel biomass had a reasonably high level of connectivity and a high degree of fragmentation respectively, owing to major wildfire events in 2003 and 2007 ([Keeley *et al.*, 2004](#_ENREF_43); [Keeley *et al.*, 2009](#_ENREF_44); [Keeley *et al.*, 2013](#_ENREF_45)). Fuel layers were obtained from the LANDFIRE database (<http://www.landfire.gov/>, accessed 10^th^ October 2012). Our study utilised fuel data layers with the classification of Scott and Burgan ([2005](#_ENREF_78)). Shrub fuel types with high fuel loading (Fuel models 145 and 147) were substituted with a shrub fuel model (Fuel model 4) from Anderson ([1982](#_ENREF_3)), as this fuel model has been shown to produce more accurate rate of spread and flame length predictions than the Scott and Burgan ([2005](#_ENREF_78)) models when tested using experimental fires ([Stephens *et al.*, 2008](#_ENREF_82)). Layers of slope, aspect and elevation were obtained from the LANDFIRE database (<http://www.landfire.gov/>, accessed 10^th^ October 2012).

**Fue**l **breaks**

Fuel breaks within southern California are typically areas of shrubland that have been converted to grasslands or bare ground, hence their capacity to stop fires is dependent upon fuel break maintenance and the presence of sufficiently equipped fire suppression crews ([Syphard *et al.*, 2011b](#_ENREF_86)). The version of FARSITE used in our study does not have the capacity to simulate fire suppression activities. To overcome this limitation we modelled fuel breaks as areas in the landscape with no fuel with a width of 90 m as this has been suggested as an appropriate width for primary fuel breaks in southern California ([Green, 1977](#_ENREF_34)). Therefore, all fuel breaks were assumed to be maintained at an optimal width and manned with sufficient fire suppression resources required to suppress fire at the fuel break. Fires could jump a fuel break provided spotting exceeded the fuel break. All mapped fuel breaks were used in the simulation ([Syphard *et al.*, 2011b](#_ENREF_86); [Syphard *et al.*, 2011c](#_ENREF_90))s, representing an optimistic scenario that assumes sufficient resources for fire suppression are available at all fuel breaks when they encounter a fire. Nevertheless, not all fuel breaks would have been included, as they may not have been mapped.

**Ignitions**

One hundred ignition locations were randomly generated across the study area. The small number of ignitions required some constraints to be placed on ignition location to facilitate fire growth and allow for the effects other simulation variables (i.e. weather and fuel characteristics) on fire size and distance spread to be properly examined. Ignitions were constrained so that they did not occur within 50 m of areas of no fuel to prevent fires from extinguishing immediately after ignition. For the same reasons, ignitions were not located within 10 km of the study area boundary and within 5 km of significant urban areas i.e. areas representing large areas without fuel. Each ignition was simulated independently, resulting in the simulation of 12000 fires (15 weather streams x 2 fuel layers x 2 LFM x 2 fuel break scenarios x 100 ignitions).

**Weather**

Three categories of fire weather were examined in our study, Santa Ana, moderate and low fire weather. The occurrence of large extreme fire events in southern California is strongly associated with the occurrence of Santa Ana winds ([Keeley *et al.*, 2009](#_ENREF_44); [Moritz *et al.*, 2010](#_ENREF_58); [Keeley *et al.*, 2012](#_ENREF_40)). A Santa Ana wind event was considered as any day during which four hourly wind speed records exceeded 32 kmh^-1^, wind direction was predominantly from the northeast and relative humidity at 1400 – 1500 hrs was less than 40% (as defined by Sergius and Huntoon ([1956](#_ENREF_79))). Fosbergs Fire Weather Index (FFWI), which is an index calculated based on temperature, relative humidity and wind speed ([Fosberg, 1978](#_ENREF_28)), was used in our study to classify the severity of non-Santa Ana fire weather. FFWI was used as there is a reasonable correspondence between FFWI and the occurrence of large fires in the south west USA ([Preisler *et al.*, 2008](#_ENREF_70)) and southern California ([Moritz *et al.*, 2010](#_ENREF_58)). Categories of 'low' and 'moderate' fire weather were defined as days when maximum FFWI was < 25 and 25 – 40 respectively. Five replicate weather streams, each selected from different fire events, were selected for each of the three weather categories (i.e. 15 individual weather streams in total). For the 'low' and 'moderate' fire weather categories, historical wildfire events were randomly selected and maximum daily FFWI was calculated, until five replicates were obtained for each category. Five known Santa Ana fire events occurring in the San Diego region in the 2003 (Cedar fire), 2007 (Poomacha, Witch and Harris 2 fires) and 2008 (Shockey fire) fire seasons were selected. The 2007 Santa Ana fires used in this study burnt during the same Santa Ana wind event (i.e. 21^st^ – 23^rd^ of October 2007), though weather streams were not taken from the same day and three different weather stations with greater than 20 km separation were used. Weather data was obtained from RAWS weather stations (<http://www.raws.dri.edu/>, accessed September – October 2012) with sufficient data that fell within 10 km of the boundary of the fire event. Weather streams commenced 5 days prior to the fire ignition to allow sufficient time for dead fuel moisture to be calibrated. Fires were ignited at 1000 hours for ‘low’ and ‘moderate’ fire weather scenarios and 0200 hours for fires burning under Santa Ana conditions. All fires were allowed to burn for a 12 hour period. The different ignition times were selected to reflect the different patterns in fire weather and enable simulations to encompass the typical peak fire weather conditions for the different fire weather categories. FFWI generally increased rapidly between 0800 and 1000 hours and reached a peak between 1000 and 1600 hours for the non-Santa Ana weather streams (L. Collins, unpublished data), while Santa Ana winds tend to be stronger in the early morning and weaken during the day ([Raphael, 2003](#_ENREF_75)).

Table S1: Fuel moisture conditions used in the simulations. Dead fuel moisture values are from Scott and Burgin ([2005](#_ENREF_11)). See text for description of LFM categories.

| **Fuel type** | **Average live fuel moisture conditions (ALFM)** | **Low live fuel moisture conditions (LLFM)** |
| --- | --- | --- |
| 1 – hr (%) | 6 | 3 |
| 10 – hr (%) | 7 | 4 |
| 100 – hr (%) | 8 | 5 |
| Live herbaceous (%) | 60 | 30 |
| Live woody (%) | 90 | 60 |
